# Fibrosis quantification using multiphoton excitation imaging of picrosirius red stained cardiac tissue

**DOI:** 10.21203/rs.3.rs-3329402/v1

**Published:** 2023-09-13

**Authors:** Bryce A. Jones, Belen Torrado, Komuraiah Myakala, Xiaoxin X. Wang, Priscilla E. Perry, Avi Z. Rosenberg, Moshe Levi, Suman Ranjit

**Affiliations:** 1 Department of Pharmacology and Physiology, Georgetown University, Washington, DC; 2 Laboratory for Fluorescence Dynamics, Department of Biomedical Engineering, University of California, Irvine; 3 Department of Biochemistry and Molecular & Cellular Biology, Georgetown University, Washington, DC; 4 Department of Pathology, Johns Hopkins School of Medicine, Baltimore, MD; 5 Microscopy and Imaging Shared Resources, Georgetown University, Washington, DC

## Abstract

Traditional methodologies for fibrosis quantification involve histological measurements, staining with Masson’s trichrome and picrosirius red (PSR), and label-free imaging using second harmonic generation (SHG). The difficulty of label-free cardiac SHG imaging is that both collagen (i.e., collagen 1 fibrils) and myosin are harmonophores that generate SHG signals, and specific identification of either collagen or myosin is difficult to achieve. Here we present an alternate method of quantifying cardiac fibrosis by using PSR staining followed by multiphoton excitation fluorescence imaging. Our data from the deoxycorticosterone model of cardiac fibrosis shows that this imaging method and downstream analyses, including background correction, are robust and easy to perform. These advantages are due to the high signal-to-noise ratio provided by PSR in areas of collagen fibers. Furthermore, the hyperspectral and fluorescence lifetime information of PSR-stained area of fibrosis shows better quantification can eventually be obtained using more complex instrumentation.

## Introduction

Heart diseases often cause the deposition of extracellular matrix proteins, including collagen, a detrimental process termed cardiac fibrosis^1^. Because heart diseases of many etiologies all converge upon cardiac fibrosis, interventions that reduce fibrosis are the subject of intense research^2^. Preclinical studies typically quantify cardiac fibrosis by transmitted light microscopy (TLM) of absorptive stains such as picrosirius red (PSR), Masson’s trichrome, and complex polychromatic stains^3,4^. However, this approach has inherent limitations which make it suboptimal.

Herein, we quantify cardiac fibrosis using two-photon fluorescent microscopy (FM) of PSR-stained rat hearts from the deoxycorticosterone acetate (DOCA) model of cardiac fibrosis^5,6^. Our approach is not affected by several limitations inherent to TLM of absorptive stains. We complement the two-photon FM with conventional TLM, second harmonic generation (SHG) imaging, third harmonic generation (THG) imaging, phasor approach to fluorescence lifetime microscopy imaging (Phasor-FLIM), and phasor approach to hyperspectral imaging (SP-Phasor). These techniques allow for additional quantification and support the robustness of our method.

The traditional approach to quantifying cardiac fibrosis with absorptive stains has several notable limitations. Absorptive staining intensity has a very narrow linear range, so it is typically quantified by calculating the percentage of positively stained tissue, not integrated intensity. This method cannot account for differences in collagen fiber density^7^, the possibility of which should not be excluded, especially if investigating antifibrotic or fibrolytic drugs. Another limitation of the traditional method is the absence of functionality in three dimensions (3D). The images are acquired on transmission light microscopes without Z-sectioning, a feature of confocal and two-photon FM.

Newer methods have been developed to circumvent these limitations. Fluorescent-based assays, such as collagen-binding probes^8–10^ and fluorogenic immunostaining^11^, scale linearly with fiber density over a wide dynamic range. Thus, they are quantitative so long as the detector is not saturated and instrument parameters are kept constant during the experiment^12–14^. Additionally, these stains can be imaged at high resolution in 3D with confocal microscopy. However, they require protocol optimization with expensive consumables and suffer from low signal-to-noise ratios due to autofluorescence of formalin-fixed paraffin-embedded (FFPE) tissues^15^. Additionally, the specificity of binding probes and antibodies is less established than traditional stains, some of which have been used for over 100 years^16–18^.

Our approach to circumvent these problems combines two-photon FM and the well-characterized histological stain PSR, first reported 60 years ago^18^. With this method, signal intensity scales linearly with dye abundance and axial sectioning allows for measurements in 3D. Additionally, unlike widefield or single-photon confocal FM of PSR^19^, two-photon FM is much less likely to prone to photobleaching. This is especially important for quantitative measurements because the common mounting medias used for absorptive stains are not optimized to prevent photobleaching of fluorescent compounds. Two-photon FM is now widely available in research laboratories and core microscopy facilities, making this method easily accessible to investigators.

The method described above arose out of difficulties we encountered while performing SHG imaging on cardiac tissue. SHG imaging identifies highly ordered, non-centrosymmetric structures, and it is often used as a method of collagen quantification in unstained tissue^20–22^. However, the data we present herein show that collagen quantification by SHG is especially difficult in cardiac tissues because both myosin and collagen are SHG active^23,24^. Thus, the natural selectivity that allows SHG to be a quantitative technique for imaging collagen in other tissues cannot be readily applied in cardiac tissues.

THG is another harmonic generation imaging method where the signal is generated by changes in refractive index between different parts of the sample. This signal can be enhanced using chemical harmonophores, and this can allow for identification of structures^25^. Recently it has been shown that PSR is a harmonophore that enables THG imaging of collagen in skin and liver^26^. In this work, we show that equivalent data can be obtained from two-photon FM and THG imaging of PSR-stained cardiac tissue, although the latter requires much more complex instrumentation and analysis.

A robust method of performing SHG and THG imaging is to acquire FLIM images and process them via Phasor-FLIM. The details of this method have been described elsewhere^27–29^. Briefly, FLIM is performed exciting the sample with a pulsed laser and recording the time required for the molecule to emit light and relax back to the ground state from the excited state achieved by absorption of the excitation light. The time the molecule stays at the excited state is termed the fluorescent lifetime. SHG and THG signals emits instantaneously and have lifetimes of zero^30^. By contrast, there is a delay before detection of fluorescence signal, represented by a non-zero fluorescence lifetime. Therefore, when performing SHG or THG, all data with a non-zero lifetimes are disregarded, isolating only the SHG or THG signal. SHG and THG signals are separated from one another by modulating the wavelength of the incident light and using filter cubes. Once FLIM data is acquired, the removal of data with non-zero lifetimes is accomplished with Phasor-FLIM.

Phasor-FLIM is a fit-free graphical method for analyzing FLIM data from individual pixels of an image, where fluorescence decays from each pixel and is transformed to a phasor plot by a Fourier transformation. Fluorescence lifetime increases in the counterclockwise direction along the phasor plot, starting at zero nanoseconds at (1,0) and approaching infinite nanoseconds at (0,0). Mono-exponential lifetimes appear on the semi-circle and lifetimes that is derived from multiple exponentials appear inside the semi-circle^31^. Pixels with similar decay times cluster together in the same region of the phasor plot, producing a phasor cloud. This allows for the easy identification of different areas of the sample with different lifetimes. SP-Phasor is a similar approach used with fluorescence spectra, where spectra acquired at each pixel are transformed to the phasor plot, and pixels of different spectra are segregated in different areas of the phasor plot.

In both Phasor-FLIM and SP-Phasor, one of the most important properties of phasors, called the reciprocity principle, is valid. According to this principle, different locations in the images can be selected, and their properties (lifetime if Phasor-FILM, spectra if SP-Phasor) can be identified on the phasor plot. Similarly, different areas of the phasor cloud distributions can be selected on the phasor plot, and the areas of the image from which the phasor data originates from can be identified. This reciprocity principle makes the analysis of FLIM and spectral data intuitive and instantaneous. In our earlier work, we have used Phasor-FLIM to isolate SHG and THG data from fluorescence data^32,33^.

In the current publication, we show the difficulty that arises with cardiac SHG imaging can be circumvented by fluorescence imaging of PSR stained cardiac tissues. Finally, we report the hyperspectral and fluorescent lifetime behavior of fibrotic regions in PSR-stained hearts, and we show that better identification of these regions is feasible using the phasor approach. Altogether, these experiments demonstrate new imaging modalities with applications to fibrosis research.

## Results

### Second harmonic generation microscopy on unstained cardiac tissue

**Figure 1.**
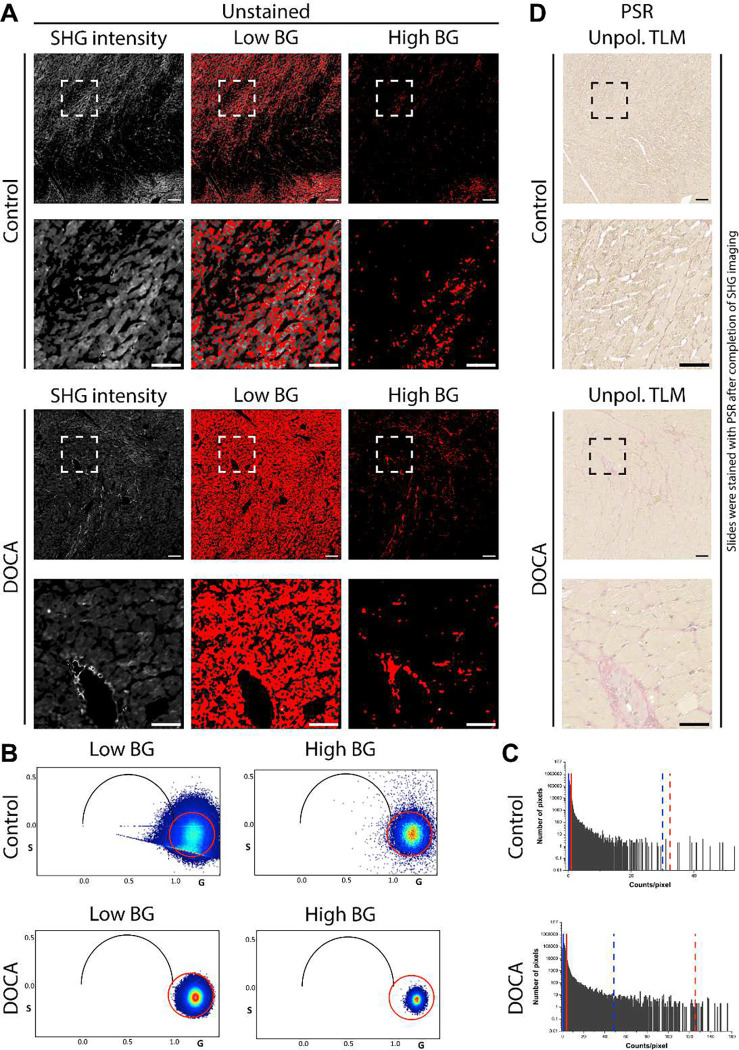
SHG of unstained rat hearts. (**A**) SHG microscopy of unstained tissue. The second row shows the zoomed-in images of the dashed regions. The SHG intensity images were color-coded red for the harmonic generation signature identified by the phasor plot. (**B**) Phasor plots of the SHG intensity image after low and high BG corrections. Data in the phasor clouds correspond with the red pixels in (A). (**C**) Histograms showing the upper (dashed) and lower (solid) thresholds used to perform the low (blue) and high (red) BG corrections. (**D**) After completion of SHG imaging on the unstained slides, they were then stained with PSR to visualize regions of fibrosis. Scale bars represent 100 μm (full images) and 50 μm (inset images). Images in Fig. 1 and Fig. 2 are from the same region of serial sections. Abbreviations: BG (background), DOCA (deoxycorticosterone), SHG (second harmonic generation), TLM (transmitted light microscopy).

**Figure 2.**
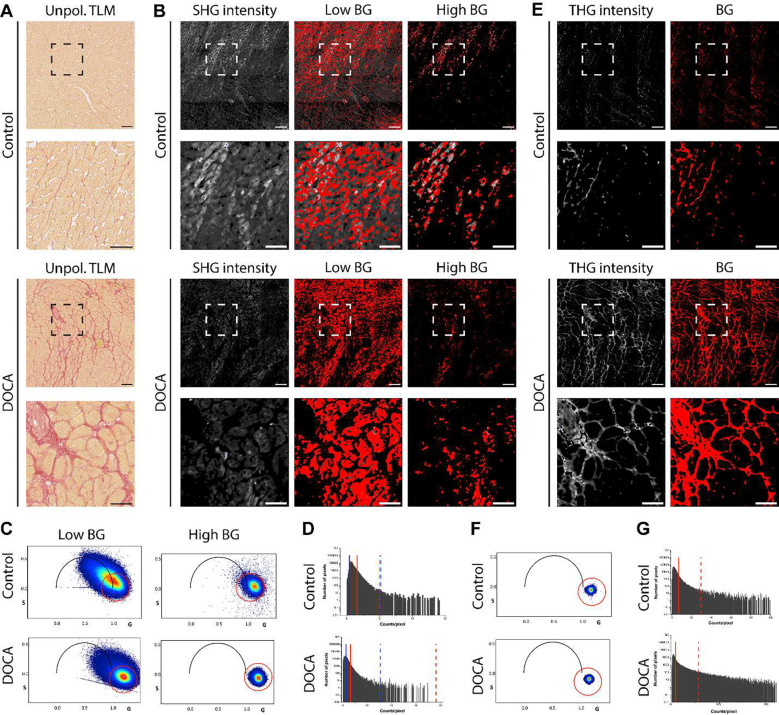
Comparison of SHG and THG in PSR-stained rat hearts. (**A**) Unpolarized TLM images form control and DOCA rat hearts. (**B**) SHG intensity images and phasor mapped images for the SHG signatures selected by the phasor plots (C) and histograms (D). Increasing the BG correction reduces, but does not eliminate, SHG signal from non-collagenous areas. (**C**) Phasor plots corresponding to the red pixels in the low BG and high BG images in (B). Increasing the BG correction isolates SHG signal and reduces bleed-through fluorescence, as shown by the phasor cloud shrinking to s=0, g=1. (**D**) Histograms showing the upper (dashed) and lower (solid) thresholds used to perform the low (blue) and high (red) BG corrections in (B). (**E**) THG images from PSR stained tissues are much more specific for cardiac fibrosis than SHG images. (**F,G**) The phasor plots (F) and histograms (G) for the THG images are shown. Scale bars represent 100 μm (full images) and 50 μm (inset images). Images in Fig. 1 and Fig. 2 are from the same region of serial sections. Abbreviations: BG (background), DOCA (deoxycorticosterone), PSR (picrosirius red), SHG (second harmonic generation), THG (third harmonic generation), TLM (transmitted light microscopy).

Second harmonic generation can be used to image fibrillar collagens in unstained tissue^22,30,32,33^, and careful data analysis may allow for fibrosis quantification in the heart^6,34,35^, especially in areas where collagen is much more abundant than myosin. However, myosin is also SHG active^24^, and it is difficult to distinguish SHG signal arising from collagen from that arising from myosin, especially in regions where collagen is not the most abundant component of tissue (Fig. 1A), as they are similar in nature. In our data, the lifetime signature confirms that the observed signal is indeed SHG and not just bleed-through fluorescence. These data show that a very large background correction (Fig. 1A, right) can indeed show areas of cardiac fibrosis with SHG imaging. However, the process is somewhat arbitrary and prone to bias. The phasor plots) show that modifying the background correction (Fig. 1B) does not change the phasor signature, just the number of pixels in the cloud (Fig. 1C). The zero lifetime of SHG appears at s=0, g=1 of the phasor plot (Fig. 1C). It also shows the original signal is a combination of collagen and myosin, and they can only be reliably differentiated from one another in areas of very extensive scarring. After acquiring the SHG images, the slides were stained with PSR to visualize the regions of fibrosis (Fig. 1D). Images in Fig. 1 and Fig. 2 are from the same region of serial sections.

### Optical harmonic generation microscopy on PSR-stained cardiac tissue

Sirius red F3B is a harmonophore, and it thus enhances the signal-to-noise ratio of optical harmonic generation imaging^26^. Based on previous results published by Kazarine et al.^26^, we hypothesized that PSR staining would enhance both SHG and THG signal, allowing fibrosis to be quantified after a minor background correction. To test this, we imaged the same region of PSR-stained hearts with TLM, SHG, and THG. The images in Fig. 1 and Fig. 2 are from the same regions of serial sections. Unpolarized TLM images of both the control and DOCA rat hearts are shown in Fig. 2A. The results show that the vast majority of SHG signal in PSR-stained cardiac tissue arises from myosin (Fig. 2B,C). Even with a large background correction (Fig. 2D), SHG signal was only marginally specific for collagen in areas of massive scarring (Fig. 2B). This reduces sensitivity and makes the isolation of collagen-specific signal difficult. Although both SHG of unstained and PSR-stained tissue may be used to study fibrosis in cardiac tissue, only the areas of abundant scarring can be identified with marginal success.

In contrast to the SHG data, THG was highly specific for PSR stained tissue in both fibrotic and non-fibrotic hearts (Fig. 2E). The bottom row of images in Fig. 2B,E demonstrates the striking difference between SHG and THG signal. The harmonic natures of the SHG and THG signals are shown by their phasor signatures in the corresponding phasor plots (Fig. 2C,F). These results show that THG, not SHG, of PSR-stained cardiac tissue is a reliable method of fibrosis quantification. Although THG has an excellent signal-to-noise ratio, as demonstrated by the minor background correction (Fig. 2G), it is technically challenging to perform. The refractive index difference between air (η= 1.0) and glass/Permount (both η= 1.52) is much larger than the small differences that exist between the tissue’s morphological changes. Furthermore, large areas are often imaged with a low numerical aperture and low magnification air objective.

### Quantitative two-photon fluorescent microscopy

**Figure 3.**
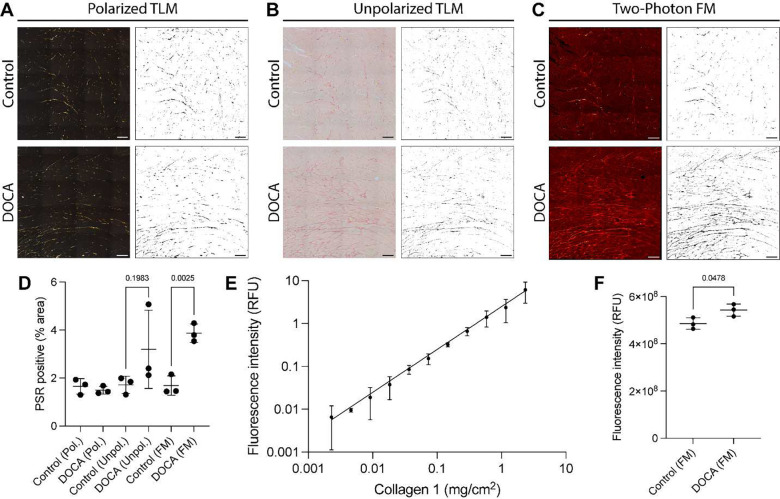
Two-photon FM of PSR-stained rat hearts. (**A-C**) Polarized TLM (A), unpolarized TLM (B), and two-photon FM (C) images of the same region of the same slide of PSR-stained control and DOCA rat hearts. (**D**) Quantification of the percent areas that are PSR-positive from 3 control and 3 DOCA hearts. Each datapoint is the average of three 1 mm^2^ regions from one rat. (**E**) Serial dilution of collagen 1 shows that Sirius red F3B fluorescence intensity is linear over at least three orders of magnitude and that it is indicative of the amount of collagen present. (**F**) Total fluorescence intensity, without background correction, from the regions in (C,D). Scale bars represent 100 μm. Significance was determined by Student’s T-test, and exact *P* values are shown. Data are expressed as the mean ± SD. Abbreviations: DOCA (deoxycorticosterone), FM (fluorescence microscopy), PSR (picrosirius red), RFU (relative fluorescence units), TLM (transmitted light microscopy).

The lack of specificity of SHG imaging in cardiac tissues and the technical difficulty of THG imaging led us to explore two-photon FM as an alternative method to quantitate cardiac fibrosis. We chose to use two-photon FM due to its simplicity and reduced likelihood to photobleach fluorescent compounds compared to widefield FM and confocal FM^19^. Polarized TLM, unpolarized TLM, and two-photon FM were performed on the same regions of control and DOCA hearts, and the percent area of PSR positive tissue was calculated (Fig. 3A–C). Importantly, comparisons between imaging methods can be made because identical regions of the same slide were imaged with all three methods. Of the three imaging techniques, two-photon FM showed the best separation between control and DOCA groups (Fig. 3D).

A known advantage of fluorescent microscopy is that signal intensity usually scales linearly with dye abundance over a wide dynamic range. This enables data to be quantified as integrated intensity as opposed to just percent area. We first verified that the fluorescence intensity of Sirius red F3B was linear over a wide range of collagen 1 surface densities (Fig. 3E). Upon quantification of integrated intensity, the difference in cardiac fibrosis between control and DOCA hearts is maintained, even without a background correction (Fig. 3F). In summary, these data show how we can quantitate fibrosis from two-photon FM of PSR stained cardiac tissue.

**Figure 4.**
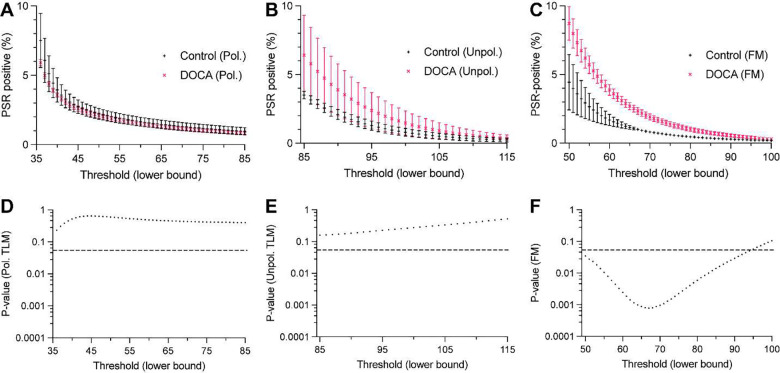
Effect of BG correction on PSR positive area of PSR-stained rat hearts across different microscopy techniques. (**A-C**) Percent area of PSR positive tissue from BG corrections that accurately segmented cardiac fibrosis in PSR-stained control (black) and DOCA (pink) rat hearts. Data were acquired with polarized TLM (A), unpolarized TLM (B), and two-photon FM (C). (**D-F**) *P* values from Student’s T-tests comparing control and DOCA groups in (A-C). Dashed line indicates *P* = 0.05. Unlike two-photon FM (C,F), neither polarized TLM (A,D) nor unpolarized TLM (B,E) identified a statistically significant increase in cardiac fibrosis in the DOCA hearts. Significance was determined by Student’s T-test. Data are expressed as the mean ± SD. Abbreviations: BG (background), DOCA (deoxycorticosterone), FM (fluorescence microscopy), PSR (picrosirius red), TLM (transmitted light microscopy).

A potential concern with the data presented in Fig. 3A–D is the effect of the background correction. Depending on the histogram correction, different amounts of signal will be segmented, and this will affect the quantification. To determine if the observed superiority of two-photon FM compared to TLM was simply due to the background correction, we calculated the percent area of PSR positive tissue for all 256 possible background corrections of the 8-bit images (Fig. 4A–C). *P* values for the comparison between control and DOCA hearts for each background correction are also plotted (Fig. 4D–F). Cardiac fibrosis in DOCA hearts was consistently increased compared to control hearts over a wide range of background corrections when measured by two-photon FM. This contrasts with both polarized and unpolarized TLM where not a single background correction was statistically significant. Background corrections that did not accurately segment PSR positive pixels are not shown. In general, this occurred when the percent area of PSR positive tissue was approximately 10%. In summary, these data show that the difference in cardiac fibrosis is only evident in the two-photon FM images, irrespective of background correction.

### Spectral and lifetime characteristics of the PSR stained tissue

**Figure 5.**
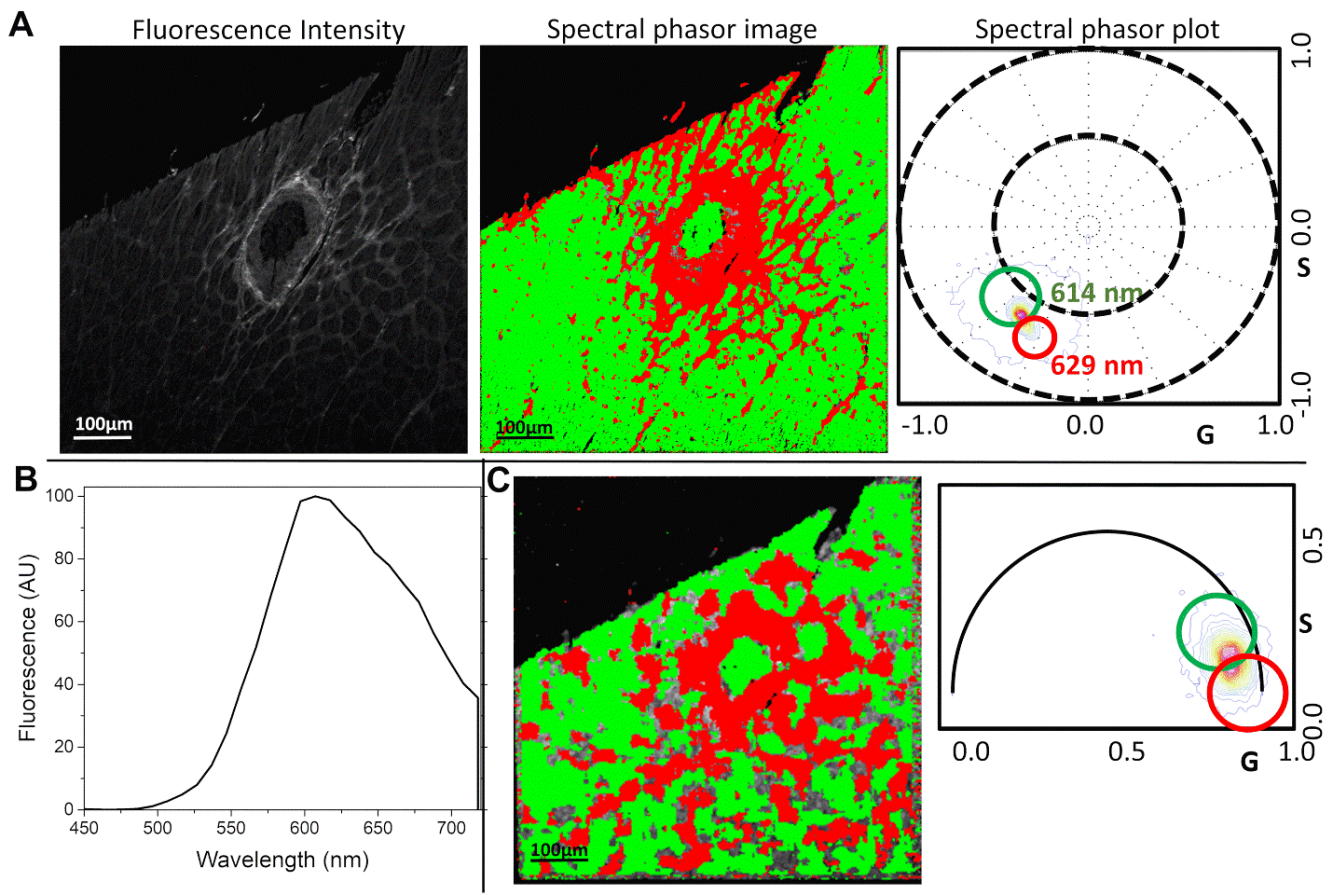
Spectral and lifetime characteristics of PSR-stained rat hearts. (**A**) Fluorescence intensity image (left) and the corresponding SP-Phasor image (center) and plot (right). The green and red pixels the SP-Phasor image correspond to the data within the green (614 nm) and red (629 nm) phasor clouds of the SP-Phasor plot, respectively. The results show that the spectral signature of the fibrotic regions is redshifted compared to non-fibrotic regions. (**B**) Fluorescence spectra of the PSR-stained cardiac tissue shows long wavelength fluorescence. (**C**) Phasor-FLIM image (center) and the corresponding phasor plot (right). The phasor plot shows that the signature of the fibrotic regions has a shorter lifetime compared to non-fibrotic regions. The Phasor-FLIM image in (C) has reduced resolution because the FLIM detector performance diminishes with longer wavelength and the data is median filtered. Scale bars represent 100 μm. Abbreviations: FLIM (fluorescence lifetime microscopy imaging), Phasor-FLIM (phasor approach to FLIM), PSR (picrosirius red), SP-Phasor (phasor approach to hyperspectral imaging).

To better understand the spectral and lifetime characteristics of PSR-stained hearts, we performed hyperspectral fluorescence imaging and FLIM at the Laboratory for Fluorescence Dynamics at the University of California, Irvine (Fig. 5). The fluorescence emission spectrum of PSR-stained cardiac tissue has a maximum above 600 nm (Fig. 5B). Both imaging modalities require specialized detectors, capable of detecting photon acquisition above 600 nm, as well as two-photon FM. FLIM and hyperspectral data were transformed to a phasor plot for analysis. The spectral phasor data (Fig. 5A) of PSR-stained cardiac tissue show that the areas of cardiac fibrosis have a spectral phasor signature with a maximum at 629 nm. This is redshifted from non-fibrotic areas that have a maximum at 614 nm. Phasor-FLIM data shows that areas of collagen accumulation have shorter lifetimes than non-collagenous areas in PSR-stained cardiac tissue (Fig. 5C). The fluorescence emission spectra of the PSR stained cardiac tissue above 600 nm is the reason for the low signal-to-noise ratio of the FLIM data^36^. This low ratio is associated with broadening the phasor clusters in the Phasor-FLIM, which requires more filtering to analyze the data, lowering the resolution of the Phasor-FLIM images.

## Discussion

Fibrosis is the common endpoint of many types of chronic diseases, including heart diseases. TLM using absorptive stains for fibrosis and scoring by a pathologist will remain the preferred method for guiding patient treatment for the foreseeable future. However, preclinical and translational research should consider alternative methods when appropriate. Our approach combines the well-validated PSR stain with two-photon FM, allowing for better quantification of cardiac fibrosis when compared to both polarized and unpolarized TLM. This approach arose from the difficulties we experienced when imaging fibrosis in unstained cardiac tissues with SHG. Specifically, collagen and myosin fibers are both SHG active, and distinguishing between them is extremely difficult, especially in areas of modest scarring.

We and others have previously shown that SHG can be used to study fibrosis in other tissues, including the kidney and liver^20–22,30,32,33^. We want to emphasize that SHG can indeed separate collagen from myosin in cardiac tissues where extensive scarring is present^6,34,35^, although the data presented herein shows that it requires careful background separation. In our experiment, Phasor-FLIM confirmed that the background signal arising from myosin is true SHG signal, not just bleed-through fluorescence. Thus, the signal arising from myosin cannot be distinguished from that of collagen by their different spectral and lifetime signatures. This difficulty in separating the SHG signal arising from collagen from that of myosin is the main reason why we explored alternative imaging techniques.

Our data show that both THG and two-photon FM can be used for fibrosis quantification in PSR-stained cardiac tissues. Further analysis, including collagen alignment and feature extractions like diameter, is possible. In this work, we have used a 20X objective for large-area imaging. Higher magnification objectives with higher numerical aperture could be used to identify finer details of the collagen structure. Additionally, THG and two-photon FM allow for 3D imaging with high Z resolution. This is not possible with traditional TLM because of the lack of Z sectioning. Although both methods accurately identify fibrosis, we chose to focus on two-photon FM because it is widely available at research institutions. THG requires specialized instrumentation and training that is difficult for most researchers to access.

Finally, we quantified the hyperspectral and fluorescent lifetime behavior of PSR-stained cardiac tissues and show that better separation is feasible using the phasor approach. FLIM imaging in this red wavelength range is still not very common due to limitations of the current GaAsP detectors. For spectral imaging, the most commonly used systems that are commercially available have a full range of 410 nm to 696 nm^37^. Hyperspectral imaging will be even better when commercial instruments allow for zooming in on the red part of the spectrum. We believe hyperspectral and FLIM imaging of cardiac fibrosis will be more common once the instrumentation becomes easily accessible.

## Methods

### Animal model

This study is reported in accordance with ARRIVE guidelines^38^. Animal experiments were performed at Georgetown University in accordance with the Association for Assessment and Accreditation of Laboratory Animal Care international guidelines and approved by the Georgetown University Institutional Animal Care and Use Committee. Animals were maintained on a 12-hour light/dark cycle and fed PicoLab Rodent Diet 20 (LabDiet, St. Louis, MO) *ad libitum*. Ten-week-old male Sprague-Dawley rats (*n* = 12) were obtained from Hilltop Lab Animals (Scottdale, PA). Six rats were unilaterally nephrectomized, implanted with 75 mg 60-day continuous-release DOCA pellets (Innovative Research of America, Sarasota, FL), and given salt-enriched drinking water (0.9% NaCl, 0.2% KCl). Unilateral nephrectomy and DOCA pellet implantation were performed by the vendor prior to shipment. Rats were sacrificed at 18 weeks by CO_2_ inhalation and the tissues were collected accordingly. Hearts were drop fixed in 10% neutral buffered formalin (NBF). One half of the rats from each group were randomly chosen for this study. The experiment was performed in an unblinded manner.

### Histological staining

Hearts were processed by the Reference Histology Laboratory at Johns Hopkins University and the Histology and Tissue Shared Resource at Georgetown University. Routine PSR staining of sectioned hearts was performed by Histoserv, Inc. (Germantown, MD). Slides were scanned with an Aperio GT 450 (Leica Biosystems, Deer Park, IL).

### Instrumentation

Conventional and polarized transmitted light microscopy (TLM) was performed using a transmission light microscope (Olympus model IX83, Waltham, MA) equipped with a 40X air immersion objective. All images from each technique were obtained with the same acquisition settings. Three 1 mm^2^ areas from each sample were imaged. The locations imaged were the exact same as those imaged with multiphoton fluorescence excitation imaging. Images were processed and quantified using QuPath and ImageJ^39,40^. Images were converted to 8-bit monochrome images, and a threshold was applied to identify PSR-positive pixels. The maximum threshold value was held constant at 255, and the minimum threshold value was incrementally increased from 0 to 255.

Multiphoton fluorescence excitation imaging was carried out using the Olympus FVMPE-RS (Waltham, MA) upright microscope equipped with an Insight X3 laser (Milpitas, CA). The samples were excited with 1040 nm laser line, using a 20X air immersion objective (Olympus, 0.45NA, Air, LUCPLFN) and the images were taken using a 575–645 nm bandpass filter. The laser frequency was 80MHz and the zoom was set as 2.5, resulting in a pixel size of 0.249 μm. The laser transmissivity was 4% and the pixel dwell time was 20 μs/pixel. The signals were collected using two non-descanned PMTs (GaAsP, 450 V PMT voltage). All images were taken with the same acquisition settings. Three 1 mm^2^ areas from each sample were imaged. The locations imaged were the exact same as those imaged with polarized and unpolarized TLM. Fluorescence images were processed and quantified using QuPath and ImageJ^39,40^. Images were converted to 8-bit monochrome images, and a threshold was applied to identify PSR-positive pixels. The maximum threshold value was held constant at 255, and the minimum threshold value was incrementally increased from 0 to 255.

Hyperspectral and fluorescence lifetime imaging was performed on a Zeiss LSM 710 microscope (Carl Zeiss, Jena, Germany) using a 20X, 0.8 NA air immersion objective, (Carl Zeiss, Oberkochen, Germany) coupled to an 80 MHz multiphoton MaiTai Ti-Sa laser (Spectra-Physics, Mountain View, CA). The samples were excited at 1020 nm. The scan speeds for hyperspectral and FLIM imaging were 6.3 μs/pixel and 12.61 μs/pixel, respectively. The images were taken with 1024X1024 and 256X256 pixels for hyperspectral and FLIM images, respectively. A long pass dichroic at 690 nm was employed to reflect the laser excitation to the sample and to separate excitation from emission signal.

The long wavelength of the mCherry channel after the emission is passed through a bandpass emission filter cube GFP/RFP coupled to a photomultiplier tube (H7422P-40, Hamamatsu, Japan) was used as the FLIM detection module with the RFP filter (690/50 nm) as the acquisition window on the microscope external detector port. FLIM data was collected using A320 FastFLIM (ISS, Champaign, IL). 51 frames were collected and integrated to increase the signal-to-noise ratio in FLIM analysis. The FLIM detectors have lower sensitivity in the long wavelength range, and a large number of frames are needed to separate the signal from the background^36^. SimFCS software (https://www.lfd.uci.edu/globals/, Laboratory for Fluorescence Dynamics, University of California, Irvine) was used for frequency domain FLIM data acquisition. The Phasor plot for FLIM acquisition was calibrated using Rhodamine 110 in water with known lifetime of 4.0 ns. The pixel dimension was 0.69 μm/pixel.

In hyperspectral image acquisition, the fluorescence from the sample was directed to the 32-channel spectral detector of a Zeiss 710. The full length of the spectral detector (416–726 nm) was used for the image acquisition. The hyperspectral data collected from each pixel of the image were then converted to spectral phasor using SimFCS. 16 lines were collected and integrated for a good signal-to-noise ratio in spectral-phasor analysis. The pixel dimension was 2.76 μm/pixel.

### FLIM analysis

In the phasor approach to FLIM, the fluorescence signals collected from each point of the image is transformed to the phasor space using the following transformation^27–29,41^.

gi,j(ω)=∫0T  Ii,j(t)⋅cos⁡(nωt)dt∫0T  Ii,j(t)dtsi,j(ω)=∫0T  Ii,j(t)⋅sin⁡(nωt)dt∫0T  Ii,j(t)dt

$I_{i,j}(t)$ is the intensity decay; $g_{i,j}(\omega )$ and $s_{i,j}(\omega )$ are the X and Y coordinates in the phasor plot, respectively; ω is the angular repetition associated to the frequency of the laser (ω=2πf); T is the period of the laser repetition frequency; and n is the harmonic frequency. i,j are the X and Y coordinates of a pixel of the original microscopy image, respectively. In frequency-domain measurements, the transformation to the phasor plot uses the following relations,

$$g_{i,j}(\omega )=m_{i,j}\cdot cos\left(\phi_{i,j}\right)$$

$$s_{i,j}(\omega )=m_{i,j}\cdot sin\left(\phi_{i,j}\right)$$

where, $g_{i,j}(\omega )$ and $s_{i,j}(\omega )$ are the X and Y coordinates of the phasor plot, respectively, and $m_{i,j}$ and $\phi_{i,j}$ are the modulation and phase at the image pixel i,j. The longer lifetime is represented by the larger phase angle in the phasor plot.

The phasor plots from the images were sectioned into two fractions with green and red cursors (circles), with the green cursor signifying longer lifetimes than the red cursor. The phasor plot was filtered using a 5X5 median filter for the low signal-to-noise ratio in long wavelengths due to the low sensitivity of the FLIM detector. The images were colored according to their lifetime/phasor signature with longer lifetime areas colored in green.

### Spectral Phasor Imaging

The fluorescence spectra collected from each pixel of the image were transformed to the spectral phasor plot using a Fourier transformation^27,31,42,43^. Briefly, this process involves calculation of the G and S coordinate using the following equations:

$$X\;coordinate=G=\frac{\sum_{\lambda }\;I(\lambda )\;\cdot \;cos\left(2\pi n\left(\lambda \;-\;\lambda_{i}\right)/L\right)}{\sum_{\lambda }\;I(\lambda )}$$

$$Y\;coordinate=S=\frac{\sum_{\lambda }\;I(\lambda )\;\cdot \;sin\left(2\pi n\left(\lambda \;-\;\lambda_{i}\right)/L\right)}{\sum_{\lambda }\;I(\lambda )}$$

where I(λ) is the intensity at each of the 32 channel steps of the spectrum, n is the number of the harmonic, and L is the total range of the acquired spectrum (728–416 = 312 nm). This transformation converts the spectra from each pixel to the spectral phasor plot and creates a heatmap where longer wavelengths go counterclockwise starting from the first coordinate, and increasing broadening (Full Width at Half Maximum, FWHM of the spectra) brings the points in phasor plot closer to the 0, 0 position.

The phasor plot was filtered using a 3X3 median filter for increasing the signal-to-noise ratio and decreasing the spread of phasor points. In the spectral phasor plot, the longer wavelength centered around 629 nm was selected using a red cursor, and the shorter wavelength centered around 614 nm was selected using a green cursor. The images were colored accordingly.

### PSR standard curve and Sirius red F3B spectra

Bovine collagen 1 (Gibco, Cat. No. A1064401) was serially diluted into a 96-well plate and incubated at 37 °C until dry. This generated a thin layer of collagen of known mass on the surface of each well. PSR staining was then done according to standard procedures, and fluorescence intensity (520 nm excitation, 600 nm emission) was recorded with a plate reader (PerkinElmer EnSPIRE, Waltham, MA). Signal from wells incubated in saturated picric acid (without Sirius red F3B) were subtracted from those incubated in PSR. Data are plotted as the average fluorescence versus collagen surface density, calculated as the mass of collagen divided by the surface area of the well.

### Statistical Analyses

Data were analyzed in GraphPad Prism (San Diego, CA). Control and DOCA groups were compared using Student’s T-test. Linear regression was performed on the standard curve to generate a line of best fit running through the origin. Data are presented as mean ± standard deviation.

## Data Availability

The imaging data in raw and modified formats are available from osf.io/y29fz. The spectral, FLIM and harmonic generation data is presented in ‘.r64’ format, which is calibrated data from FLIM and hyperspectral detector (Zeiss) that are referenced with calibration standard Rhodamine 110. These data can be opened and analyzed using SimFCS (https://www.lfd.uci.edu/globals/).
